# Radical Smiles Rearrangement: An Update

**DOI:** 10.3390/molecules21070878

**Published:** 2016-07-06

**Authors:** Ingrid Allart-Simon, Stéphane Gérard, Janos Sapi

**Affiliations:** Institut de Chimie Moléculaire de Reims, UMR CNRS 7312, Université de Reims-Champagne-Ardenne, Faculté de Pharmacie, 51 rue Cognacq-Jay, F-51096 Reims Cedex, France; simoningrid@hotmail.fr

**Keywords:** radical chemistry, Smiles rearrangement, domino reactions, radical cascade, heterocycle

## Abstract

Over the decades the Smiles rearrangement and its variants have become essential synthetic tools in modern synthetic organic chemistry. In this mini-review we summarized some very recent results of the radical version of these rearrangements. The selected examples illustrate the synthetic power of this approach, especially if it is incorporated into a domino process, for the preparation of polyfunctionalized complex molecules.

## 1. Introduction

The Smiles rearrangement is an intramolecular nucleophilic *ipso*-substitution on an aromatic ring system, activated with electron withdrawing group(s) at the *ortho*- and/or *para*-position(s) with respect to the reaction center [1]. In its original version the *ipso*-attack is followed by the migration of the activated aromatic ring from the heteroatom bound to the reaction center to a more nucleophilic heteroatom [2]. Substrate-scope investigations evidenced the importance of electronic and steric effects of ring substituents on the efficiency of the reaction. Over the decades the Smiles rearrangement has found widespread synthetic application in organic chemistry and different varieties of the rearrangement have been reported so far [3]. For example, in the Truce-Smiles version the nucleophile is typically a carbanion rather than a heteroatom, thus allowing the formation of carbon-carbon bonds [4].

The Smiles rearrangement and its different variants were originally developed under ionic conditions and Speckamp was the first to transpose it to radical chemistry [5,6]. Later on, Motherwell pointed out the importance of this approach, especially for the synthesis of biaryl systems [7,8,9]. In fact, the biaryl appendage is a common structural unit of many natural products, pharmacologically active compounds, chiral ligands, organic materials, or optoelectronic devices. To construct these structures radical chemistry-based methods are considered to be additional tools for synthetic organic chemists. The radical Smiles rearrangement is traditionally triggered by the attack of free radicals on the *ipso*-position of sulfonates or sulfonamides, followed by sulfur dioxide extrusion and final hydrogen abstraction, but the presence of an activating substituent in the migrating unit is not essential. It is important to note that desulfuration is sometimes replaced by a decarboxylation step.

The Smiles rearrangement has recently appeared in multi-component reactions or one-pot approaches [10,11,12], but only a few reports of domino strategies involving this rearrangement are available, especially in a radical manner [13,14,15,16].

This mini-review, focused on the radical version of the Smiles rearrangement, presents recent synthetic achievements, updating the work of Tu on radical aryl migration reactions [17]. We will first present the more widespread sp^3^C-centered radical-promoted Smiles rearrangement, while a second section will be devoted to sp^2^C radical-assisted approaches.

## 2. sp^3^ C-Centered Radical-Promoted Rearrangements

**Scheme 1:**
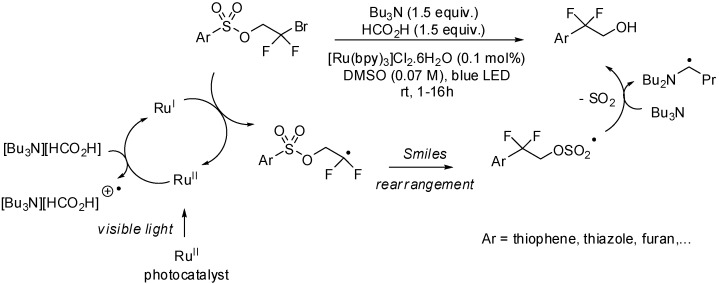
Photocatalyzed radical Smiles rearrangement of difluorobromomethyl arylsulfonates.

In the last two decades increased attention was focused on light-induced chemical transformations, especially in the field of radical chemistry [18,19]. UV or visible light can be used as green, sustainable and inexpensive sources of energy. Light limits waste in radical reactions, is environmental friendly, and makes purification of reaction products easier. In this line, Stephenson et al. have developed a visible-light-mediated radical Smiles rearrangement of difluorobromo arylsulfonates enabling a direct and efficient introduction of the difluoroethanol moiety into a range of aryl and heteroaryl ring-systems (Scheme 1) [20]. From a mechanistic point of view, the reaction starts with the quenching of the excited state of the photocatalyst ([Ru(bpy)_3_]^2+^) by a an electron donor (Bu_3_N·HCO_2_H) to give the strongly reducing Ru^I^ species. Single-electron reduction of the substrate affords the expected difluoroethyl radical that undergoes a consecutive *ipso*-addition—SO_2_ elimination—H atom abstraction process to provide the desired product.

**Scheme 2:**
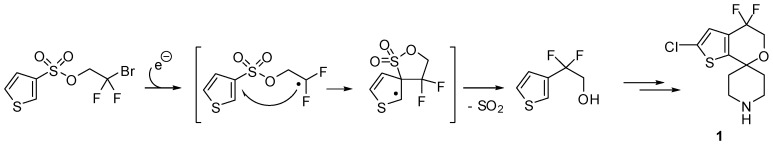
Application to the synthesis of difluoro-substituted spirocyclic ORL-1 antagonist.

This domino process was successfully applied for the synthesis of the opioid receptor-like 1 antagonist **1** used for the treatment of depression and/or obesity (Scheme 2).

**Scheme 3:**
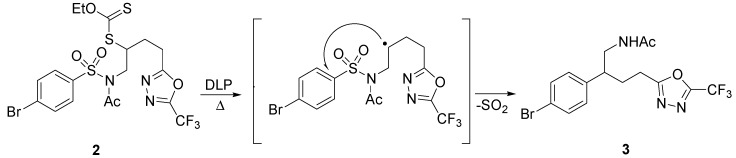
Example of Smiles rearrangement starting from xanthate.

Another example of Smiles rearrangement, triggered by generation of the radical from a xanthate with dilauroyl peroxide (DLP) was proposed by Zard, as illustrated by Scheme 3. Sulfonamide **2** furnished the corresponding trifluoromethylated oxadiazole **3** in 32% yield [21].

**Scheme 4:**
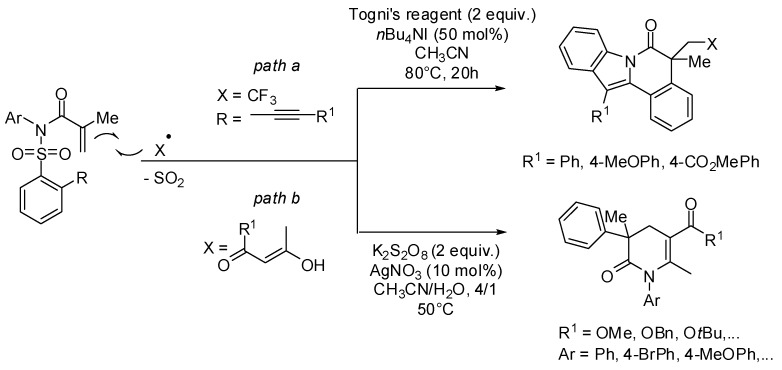
Cyclization cascades via *N*-amidyl radicals.

For some years Nevado and coworkers have been interested in the development of new domino reactions involving the addition of radicals to the double bond of *N*-(arylsulfonyl)acrylamides and subsequent radical Smiles rearrangements. In a recent work they demonstrated the synthetic utility of in-situ generated amidyl radical intermediates in additional C-C or C-heteroatom bond forming reactions affording remarkable molecular complexity [22]. Thus, they described a regioselective one-pot process for the synthesis of CF_3_, SCF_3_, P(O)Ph_2_, and N_3_-containing indolo[2,1-*a*]isoquinolin-6(*5H*)-ones of potential biological interest (path a, Scheme 4). This multi-step process consists of an addition of radical followed by *ipso*-substitution, 1,4-aryl group migration/desulfonylation, 5-*exo-trig* cyclization and final H-abstraction.

With 1,3-dicarbonyl compounds as primary radical precursors, highly functionalized dihydropyridinone-type derivatives were prepared in a silver-catalyzed radical domino reaction process (path b, Scheme 4). Dicarbonyl compounds act as both radical donors and acceptors adding to the acrylamide C=C bond and trapping the key-intermediate amidyl radical, respectively.

A systematic substrate scope study concerning the radical sources as well as each part of the *N*-(arylsulfonyl)acrylamide moiety evidenced the preparative efficiency of this multi-step radical reaction cascade.

**Scheme 5:**
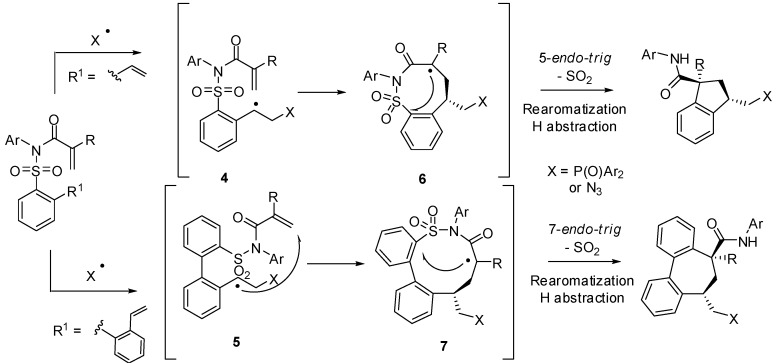
Stereoselective synthesis of highly functionalized indanes and dibenzocycloheptadienes.

When *ortho*-vinyl- or *ortho*-styryl-substituted *N*-(arylsulfonyl)arylamide substrates were reacted with carbon or heteroatom centered radicals a multistep radical domino process takes place to afford multifunctionalized indane- and dibenzocycloheptadiene-type products in a stereoselective manner (*trans* relative configuration between amide and phosphonyl or azide functions) [23]. According to the proposed mechanism (Scheme 5) primary radicals react with the vinyl moiety to give benzyl radicals **4** or **5** that undergo 8-*endo*- or 10-*endo*-*trig* cyclization. With the obtained α-keto radicals (**6** or **7**) a second 5- (or 7-membered) *ipso*-cyclization occurs provoking a subsequent 1,4-aryl group migration—desulfonylation—rearomatization—H-abstraction process, as part of a Smiles rearrangement.

**Scheme 6:**
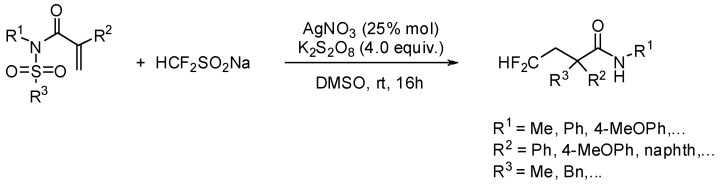
Difluoromethylation of conjuguated arylsulfonyl amides.

Using the same type of substrate Hu and *coll*. have proposed a straightforward introduction of mono- and difluoroalkyl groups via a radical process. From *N*-arylsulfonylated acrylamides and fluorinated alkylsodium sulfinate, a silver-catalyzed domino fluoroalkylation—aryl migration—SO_2_ extrusion sequence allowed the synthesis of α-aryl-β-fluoroalkyl amides in acceptable to good yields (Scheme 6) [24]. Contrary to Nevado’s findings, the corresponding 3-fluoroalkyl-2-oxindoles as minor products were observed in a few cases only. Their formation may be explained by an additional *5-exo-trig* cyclization of the intermediate amidyl radical.

**Scheme 7:**
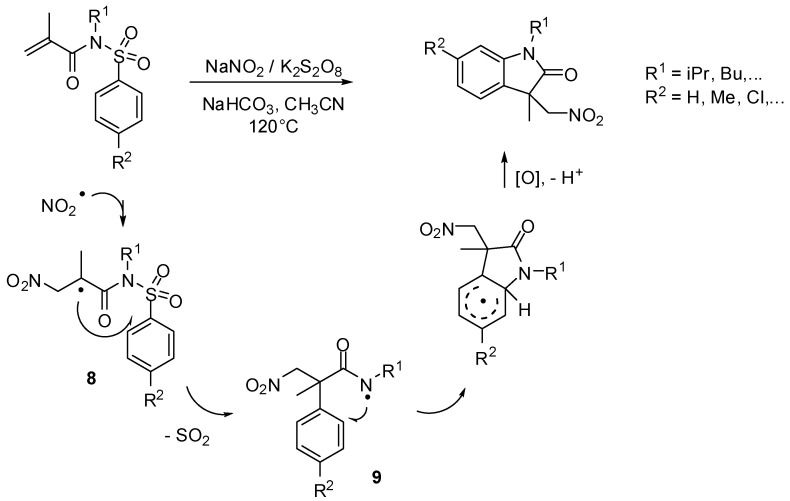
Synthesis of nitromethyl-substituted oxindole derivatives.

Nitromethyl-substituted oxindole derivatives could be obtained via a radical cascade process involving substitution—desulfonylation—cyclization steps in a one-pot procedure without using toxic metal catalysts. The procedure used NaNO_2_ as a nitro source and potassium peroxydisulfate as an oxydant (Scheme 7). According to the mechanism proposed by Zhou and coworkers, the reaction starts with the formation of a nitrogen dioxide radical that adds to *N*-alkyl-*N*-(phenylsulfonyl)acrylamide resulting in the intermediate **8**. Smiles rearrangement generates intermediates **9** that give oxindole derivatives in good yields. The obtained nitromethyl-substituted oxindoles are valuable structures for further synthetic applications [25].

In several cases Smiles-type rearrangement combined with intramolecular radical *ipso*-substitution on an aromatic group triggered structural transformations (aromatic moiety migration) without sulfur dioxide extrusion.

**Scheme 8:**
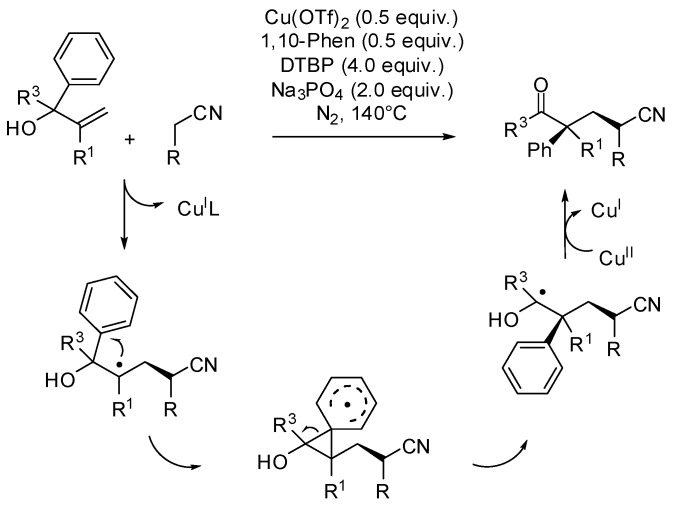
Efficient synthesis of functionalized ketones containing an α-quaternary center.

In this line α-aryl allylic alcohols have recently emerged as valuable substrates for the difunctionalization of the alkene moiety by transition-metal catalysts or environmentally friendly light catalysis. For example, Zhu et al. have developed a Cu(OTf)_2_-catalyzed radical coupling between allylic alcohols and alkyl nitriles for the synthesis of α-aryl substituted β- or γ-cyanoketones [26]. This domino process involved the formation of an alkylnitrile radical, its intramolecular addition to the C=C double bond, followed by 1,2-aryl migration and generation of a carbonyl group, as main steps (Scheme 8).

**Scheme 9:**
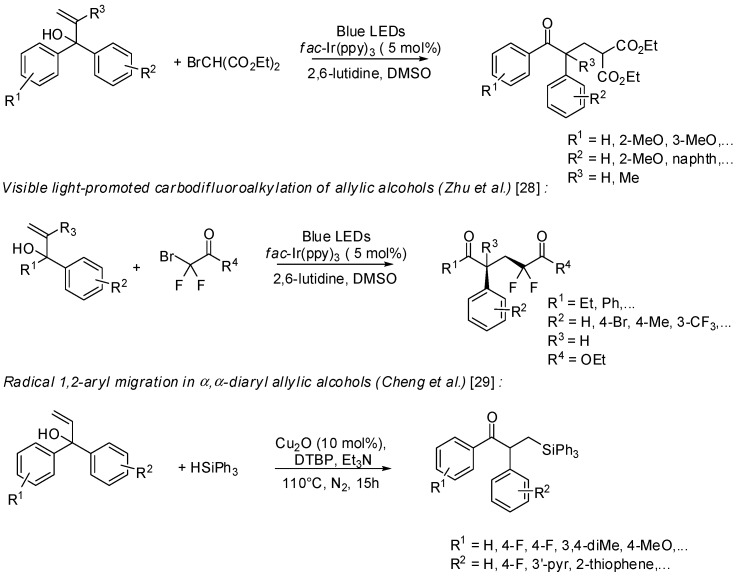
Reactivity of allylic alcohols under radical conditions.

Starting from similar allylic alcohols Xia et al. proposed a visible light-promoted radical arylalkylation with diethyl bromomalonate [27]. Optimization of the reaction conditions evidenced *fac*-Ir(ppy)_3_ as the best photoredox catalyst in DMSO, in the presence of 2,6-lutidine as additive (Scheme 9). The potential application of this approach was demonstrated by a formal synthesis of (±)-sequirin D, a norlignan-type natural product with anti-gonadotropic activity.

Very similar reaction conditions were found for the radical carbodifluoroalkylation of 1,1-diaryl allylic alcohols affording α-difluoro-γ-aryl-δ-oxoesters of synthetic interest [28]. Using CF_3_I as a relatively inexpensive CF_3_ source and Ru(bpy)_3_(PF_6_)_2_ as an alternative photoredox catalyst various α-aryl-β-trifluoromethylketones were also synthesized. Both reactions meet the increasing demand for new and efficient fluorination approaches.

Cheng and *coll*. have treated 1,1-diaryl allylic alcohols with aryl(alkyl)silanes in the presence of di(*tert*-butyl)peroxide (DTBP), triethylamine and copper(I)oxide as catalyst to give α-aryl-β-silylketones in moderate to good yields [29].

Scope and limitation studies revealed in all cases a favored migration tendency for electron deficient aryl groups affording functionalized ketones with moderate to good regioselectivities.

**Scheme 10:**
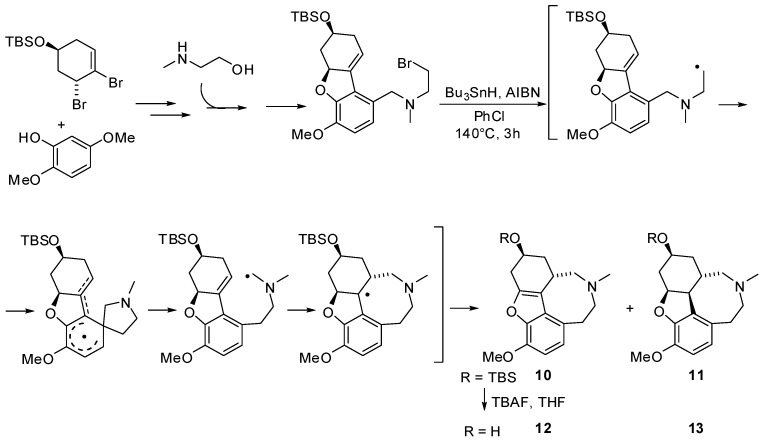
Synthesis of a D-ring isomer of galanthamine.

For the synthesis of the D-ring isomer of galanthamine, a competitive and reversible inhibitor of acetylcholine esterase, Banwell and *coll*. have exploited a seven-step process including a radical Smiles rearrangement as key-step (Scheme 10) [30]. This example demonstrates how tertiary benzylamines bearing a β-bromoethyl moiety may be involved in a radical Smiles rearrangement giving rise to a radical intermediate ready to add to a non-aromatic π-system by an 8-*endo-trig* cyclization. Contrary to expectations, eight-membered analog **12** displayed low in vitro acetylcholine esterase inhibitory potency, confirmed by subsequent molecular docking studies.

## 3. sp^2^ C-Centered Radical-Promoted Rearrangements

Motherwell is one of the pioneers who has exploited the radical version of the Smiles rearrangement as an alternative path to transition metal-catalyzed coupling reactions for the synthesis of functionalized biaryl derivatives [7].

**Scheme 11:**
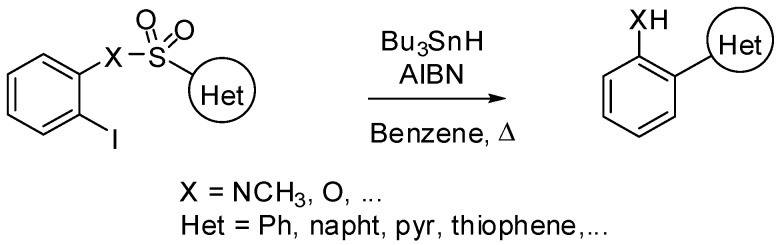
Synthesis of biaryls.

A recent paper of his group deals with a systematic scope and limitation study of this rearrangement by varying reaction conditions, the nature of the aryl acceptor ring with its substituents and the tethering chain (Scheme 11). It was found that the 1,5-*ipso*-substitution vs. 1,6-cyclization ratio was strongly impacted by the stoichiometry, the concentration, the rate of addition of Bu_3_SnH, the temperature and the solvent used in the radical reaction. *Ortho* substituents, both electron-donating and electron-attracting groups on the aromatic acceptor ring were clearly revealed to be *ipso-directors*. Similarly, substrates bearing sulfonamide-type linker afforded 1,5-*ipso* products more readily comparing to the sulfonate or carbonate analogs owing to a faster SO_2_ extrusion (vs. CO_2_) from the radical intermediate. Heteroarylsulfonates or sulfonamides submitted to optimized reaction conditions afforded mainly or exclusively 1,5-*ipso*-substitution products. Extension of this reaction to benzylic sulfonamides or sulfonates evidenced a preference for the 1,7-cyclization path even if in some cases 1,6-*ipso*-products were isolated in small quantity [31].

**Scheme 12:**
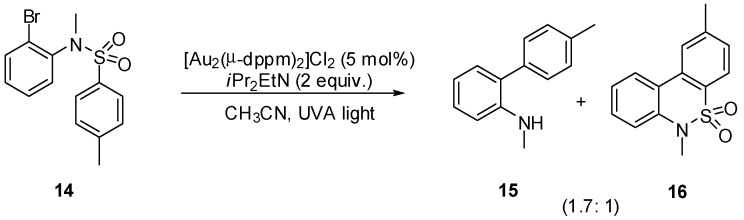
Photoredox cyclization of arylbromide using a dimeric gold complex.

As part of a study to explore new transition-metal complexes as potential photocatalysts Barriault et al. have described a light-induced reductive radical reaction of unactivated alkyl and aryl bromides using a dimeric phosphine-gold complex. Scope and limitation experiments demonstrated the potential of the catalyst both in intra- and intermolecular transformations. As expected, among the screened substrates sulfonamide **14** afforded mainly the biaryl amine **15** by *ipso*-substitution along with the tricyclic sulfonamide **16** in 93% overall yield (Scheme 12) [32].

Cascade reactions initiated by radical addition to alkynes are synthetically very attractive enabling a short access to complex polyfunctionalized derivatives, especially when they are combined with a Smiles-rearrangement.

**Scheme 13:**
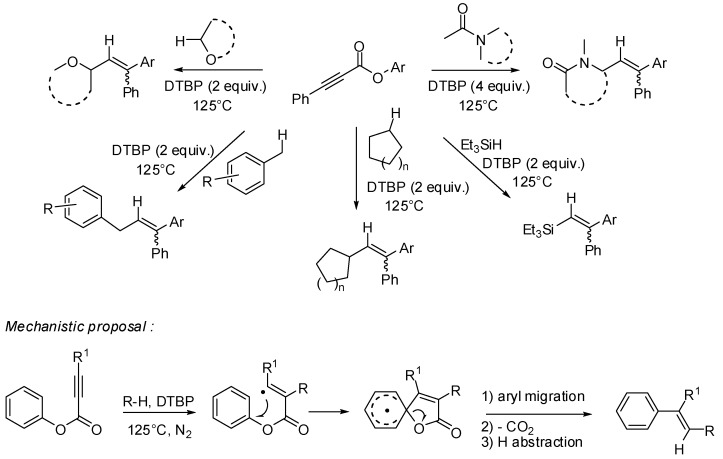
Cascade reaction of aryl alkynoates with different types of radical precursors.

Recently, Liu and *coll.* described a convenient metal-free radical yne-addition—*ipso*-substitution—decarboxylation cascade reaction of aryl alkynoates to lead to functionalized trisubstituted alkenes (Scheme 13). In these di(*tert*-butyl)peroxide-initiated reactions sp^3^ C-H substrates such as cyclic ethers, amides, benzylic hydrocarbons, cycloalkanes or triethylsilane act both as radical precursors and hydrogen donors [33].

**Scheme 14:**
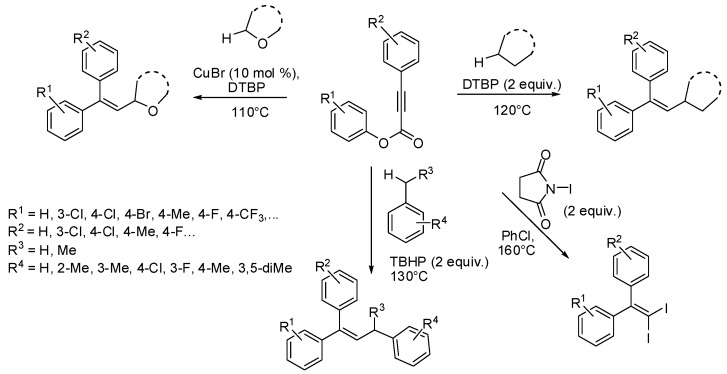
Functionalization of aryl alkynoates via radical cascade.

In parallel, the same multi-step radical cascade was also studied by Zhu [34], Wang [35], and Pan [36] using cyclic or acyclic ethers, toluene derivatives and cycloalkanes as sp^3^ C-H substrates and DTBP/Cu(I), *tert*-butylhydroperoxide (TBHP) and di-*tert*-butylperoxide (DTBP) as oxidants, respectively (Scheme 14). The reaction displayed broad scope and high functional group compatibility leading to a great variety of trisubstituted olefins with moderate to good yields. In addition, Pan also developed an efficient cascade trifunctionalization reaction of alkynoates with *N*-iodosuccinimide for the preparation of 1,1-diiodoalkenes through an iodination—aryl migration—decarboxylation and second iodination process [37].

**Scheme 15:**
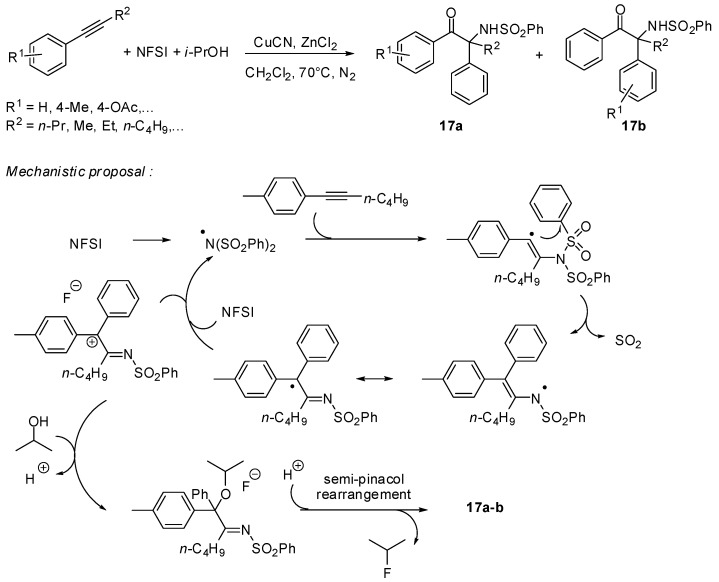
Multifunctionalization of alkynes with NFSI.

Recently, Zhang reported a radical cascade process between alkynes, *N*-fluoroarylsulfonimides and simple alcohols enabling the efficient synthesis of α-amino-α-aryl ketones **17**. This process combines four subsequent reactions: the vinyl radical formation from *N*-fluorobenzenesulfonimide, (NFSI), followed by the aryl group migration, an efficient desulfonylation and a final semi-pinacol rearrangement (Scheme 15) [38].

**Scheme 16:**
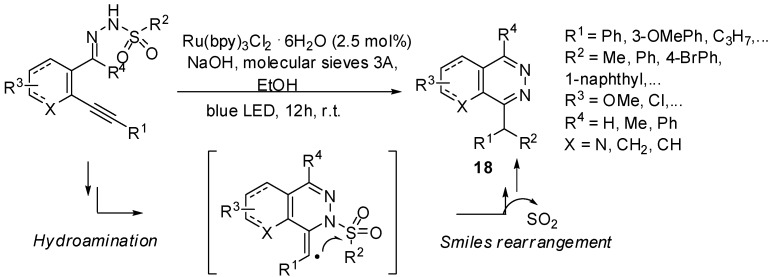
Hydroamination/Smiles rearrangement cascade.

Visible light Ru(II)-photoredox catalysis has been used by Belmont and *coll.* in a radical cascade hydroamination—Smiles rearrangement process for the synthesis of various phthalazine derivatives **18** (Scheme 16) [39]. The mild reaction conditions combined with an excellent functional group tolerance make this approach particularly attractive to reach a high molecular diversity. In addition, halogenoaryl substituents are well tolerated allowing subsequent transition metal-catalyzed functionalizations. 

**Scheme 17:**
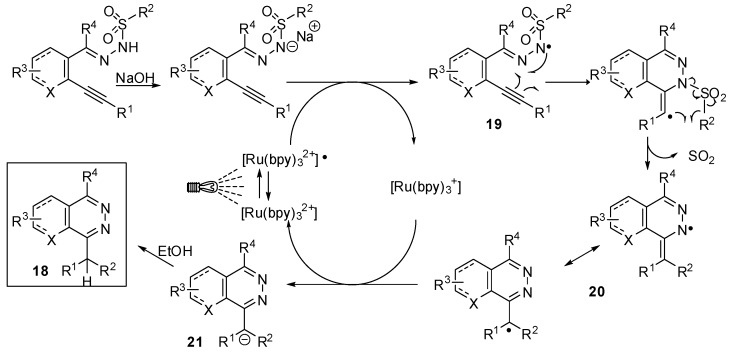
Proposed mechanism for hydroamination/Smiles rearrangement cascade.

Mechanistic investigations (Scheme 17) revealed a sodium hydroxide-mediated deprotonation and photooxidation as initial steps to give an *N*-centered radical **19**. Addition of this latter to the triple bond and SO_2_ extrusion led to the phthalazine radical **20**. The diarylmethyl anion **21** obtained by reduction of **20** was protonated (or deuterated in CD_3_OD) to afford the final product **18**.

## 4. Conclusions

During recent years, radical chemistry has played an important role in the development of modern synthetic methodologies, especially for the preparation of highly complex and polycyclic molecular frameworks. This mini-review demonstrates again that the radical Smiles rearrangement is now a well established and powerful addition to the synthetic chemistry toolbox and we are convinced that the selected examples will meet with the interest of researchers who have already been or will be engaged in this research topic.
